# Supplementary material for: Resolving the full spectrum of human genome variation using Linked-Reads
Source: Genome Res. 2019 Apr;29(4):635–45. doi: 10.1101/gr.234443.118 (PMC6442396; doi:10.1101/gr.234443.118)
Supplement: Supplemental Material [file supp_gr.234443.118_Supplemental_longranger-2.2.zip › longranger-2.2.tar.bz2!/lr2.2-public/lib/stan/cmdstan/stan/doc/api/html/cauchy_8hpp_source.html]

Stan: src/stan/prob/distributions/univariate/continuous/cauchy.hpp Source File


|  |  |
| --- | --- |
|  | Stan  2.5.0  probability, sampling & optimization |


- Main Page
- Related Pages
- Namespaces
- Classes
- Files

- File List
- File Members

All Classes Namespaces Files Functions Variables Typedefs Enumerations Enumerator Friends Macros Pages

- src
- stan
- prob
- distributions
- univariate
- continuous

cauchy.hpp

Go to the documentation of this file.

1 #ifndef STAN\_\_PROB\_\_DISTRIBUTIONS\_\_UNIVARIATE\_\_CAUCHY\_HPP

2 #define STAN\_\_PROB\_\_DISTRIBUTIONS\_\_UNIVARIATE\_\_CAUCHY\_HPP

3

4 #include <boost/random/cauchy\_distribution.hpp>

5 #include <boost/random/variate\_generator.hpp>

6

7 #include <stan/agrad/partials\_vari.hpp>

8 #include <stan/prob/traits.hpp>

9 #include <stan/math/error\_handling.hpp>

10 #include <stan/math/constants.hpp>

11 #include <stan/math/functions/log1p.hpp>

12 #include <stan/math/functions/square.hpp>

13 #include <stan/math/functions/value\_of.hpp>

14 #include <stan/math/functions/log1p.hpp>

15 #include <stan/prob/constants.hpp>

16

17 namespace stan {

18

19  namespace prob {

20

39  template <bool propto,

40  typename T\_y, typename T\_loc, typename T\_scale>

41  typename return\_type<T\_y,T\_loc,T\_scale>::type

42  cauchy\_log(const T\_y& y, const T\_loc& mu, const T\_scale& sigma) {

43  static const char\* function = "stan::prob::cauchy\_log(%1%)";

44

45  using stan::is\_constant\_struct;

46  using stan::math::check\_positive\_finite;

47  using stan::math::check\_finite;

48  using stan::math::check\_not\_nan;

49  using stan::math::check\_consistent\_sizes;

50  using stan::math::value\_of;

51

52  // check if any vectors are zero length

53  if (!(stan::length(y)

54  && stan::length(mu)

55  && stan::length(sigma)))

56  return 0.0;

57

58  // set up return value accumulator

59  double logp(0.0);

60

61  // validate args (here done over var, which should be OK)

62  check\_not\_nan(function, y, "Random variable", &logp);

63  check\_finite(function, mu, "Location parameter", &logp);

64  check\_positive\_finite(function, sigma, "Scale parameter", &logp);

65  check\_consistent\_sizes(function,

66  y,mu,sigma,

67  "Random variable","Location parameter",

68  "Scale parameter",

69  &logp);

70

71  // check if no variables are involved and prop-to

72  if (!include\_summand<propto,T\_y,T\_loc,T\_scale>::value)

73  return 0.0;

74

75  using stan::math::log1p;

76  using stan::math::square;

77

78  // set up template expressions wrapping scalars into vector views

79  VectorView<const T\_y> y\_vec(y);

80  VectorView<const T\_loc> mu\_vec(mu);

81  VectorView<const T\_scale> sigma\_vec(sigma);

82  size\_t N = max\_size(y, mu, sigma);

83

84  DoubleVectorView<true, is\_vector<T\_scale>::value> inv\_sigma(length(sigma));

85  DoubleVectorView<true, is\_vector<T\_scale>::value> sigma\_squared(length(sigma));

86  DoubleVectorView<include\_summand<propto,T\_scale>::value,

87  is\_vector<T\_scale>::value> log\_sigma(length(sigma));

88  for (size\_t i = 0; i < length(sigma); i++) {

89  const double sigma\_dbl = value\_of(sigma\_vec[i]);

90  inv\_sigma[i] = 1.0 / sigma\_dbl;

91  sigma\_squared[i] = sigma\_dbl \* sigma\_dbl;

92  if (include\_summand<propto,T\_scale>::value) {

93  log\_sigma[i] = log(sigma\_dbl);

94  }

95  }

96

97  agrad::OperandsAndPartials<T\_y, T\_loc, T\_scale> operands\_and\_partials(y, mu, sigma);

98

99  for (size\_t n = 0; n < N; n++) {

100  // pull out values of arguments

101  const double y\_dbl = value\_of(y\_vec[n]);

102  const double mu\_dbl = value\_of(mu\_vec[n]);

103

104  // reusable subexpression values

105  const double y\_minus\_mu

106  = y\_dbl - mu\_dbl;

107  const double y\_minus\_mu\_squared

108  = y\_minus\_mu \* y\_minus\_mu;

109  const double y\_minus\_mu\_over\_sigma

110  = y\_minus\_mu \* inv\_sigma[n];

111  const double y\_minus\_mu\_over\_sigma\_squared

112  = y\_minus\_mu\_over\_sigma \* y\_minus\_mu\_over\_sigma;

113

114  // log probability

115  if (include\_summand<propto>::value)

116  logp += NEG\_LOG\_PI;

117  if (include\_summand<propto,T\_scale>::value)

118  logp -= log\_sigma[n];

119  if (include\_summand<propto,T\_y,T\_loc,T\_scale>::value)

120  logp -= log1p(y\_minus\_mu\_over\_sigma\_squared);

121

122  // gradients

123  if (!is\_constant\_struct<T\_y>::value)

124  operands\_and\_partials.d\_x1[n] -= 2 \* y\_minus\_mu / (sigma\_squared[n] + y\_minus\_mu\_squared);

125  if (!is\_constant\_struct<T\_loc>::value)

126  operands\_and\_partials.d\_x2[n] += 2 \* y\_minus\_mu / (sigma\_squared[n] + y\_minus\_mu\_squared);

127  if (!is\_constant\_struct<T\_scale>::value)

128  operands\_and\_partials.d\_x3[n] += (y\_minus\_mu\_squared - sigma\_squared[n]) \* inv\_sigma[n] / (sigma\_squared[n] + y\_minus\_mu\_squared);

129  }

130  return operands\_and\_partials.to\_var(logp);

131  }

132

133  template <typename T\_y, typename T\_loc, typename T\_scale>

134  inline

135  typename return\_type<T\_y,T\_loc,T\_scale>::type

136  cauchy\_log(const T\_y& y, const T\_loc& mu, const T\_scale& sigma) {

137  return cauchy\_log<false>(y,mu,sigma);

138  }

139

140

156  template <typename T\_y, typename T\_loc, typename T\_scale>

157  typename return\_type<T\_y,T\_loc,T\_scale>::type

158  cauchy\_cdf(const T\_y& y, const T\_loc& mu, const T\_scale& sigma) {

159

160  // Size checks

161  if ( !( stan::length(y) && stan::length(mu)

162  && stan::length(sigma) ) )

163  return 1.0;

164

165  static const char\* function = "stan::prob::cauchy\_cdf(%1%)";

166

167  using stan::math::check\_positive\_finite;

168  using stan::math::check\_finite;

169  using stan::math::check\_not\_nan;

170  using stan::math::check\_consistent\_sizes;

171  using boost::math::tools::promote\_args;

172  using stan::math::value\_of;

173

174  double P(1.0);

175

176  check\_not\_nan(function, y, "Random variable", &P);

177  check\_finite(function, mu, "Location parameter", &P);

178  check\_positive\_finite(function, sigma, "Scale parameter", &P);

179  check\_consistent\_sizes(function, y, mu, sigma,

180  "Random variable", "Location parameter",

181  "Scale Parameter",

182  &P);

183

184  // Wrap arguments in vectors

185  VectorView<const T\_y> y\_vec(y);

186  VectorView<const T\_loc> mu\_vec(mu);

187  VectorView<const T\_scale> sigma\_vec(sigma);

188  size\_t N = max\_size(y, mu, sigma);

189

190  agrad::OperandsAndPartials<T\_y, T\_loc, T\_scale>

191  operands\_and\_partials(y, mu, sigma);

192

193  // Explicit return for extreme values

194  // The gradients are technically ill-defined, but treated as zero

195  for (size\_t i = 0; i < stan::length(y); i++) {

196  if (value\_of(y\_vec[i]) == -std::numeric\_limits<double>::infinity())

197  return operands\_and\_partials.to\_var(0.0);

198  }

199

200  // Compute CDF and its gradients

201  using std::atan;

202  using stan::math::pi;

203

204  // Compute vectorized CDF and gradient

205  for (size\_t n = 0; n < N; n++) {

206

207  // Explicit results for extreme values

208  // The gradients are technically ill-defined, but treated as zero

209  if (value\_of(y\_vec[n]) == std::numeric\_limits<double>::infinity()) {

210  continue;

211  }

212

213  // Pull out values

214  const double y\_dbl = value\_of(y\_vec[n]);

215  const double mu\_dbl = value\_of(mu\_vec[n]);

216  const double sigma\_inv\_dbl = 1.0 / value\_of(sigma\_vec[n]);

217

218  const double z = (y\_dbl - mu\_dbl) \* sigma\_inv\_dbl;

219

220  // Compute

221  const double Pn = atan(z) / pi() + 0.5;

222

223  P \*= Pn;

224

225  if (!is\_constant\_struct<T\_y>::value)

226  operands\_and\_partials.d\_x1[n]

227  += sigma\_inv\_dbl / (pi() \* (1.0 + z \* z) \* Pn);

228  if (!is\_constant\_struct<T\_loc>::value)

229  operands\_and\_partials.d\_x2[n]

230  += - sigma\_inv\_dbl / (pi() \* (1.0 + z \* z) \* Pn);

231  if (!is\_constant\_struct<T\_scale>::value)

232  operands\_and\_partials.d\_x3[n]

233  += - z \* sigma\_inv\_dbl / (pi() \* (1.0 + z \* z) \* Pn);

234

235  }

236

237  if (!is\_constant\_struct<T\_y>::value) {

238  for(size\_t n = 0; n < stan::length(y); ++n)

239  operands\_and\_partials.d\_x1[n] \*= P;

240  }

241  if (!is\_constant\_struct<T\_loc>::value) {

242  for(size\_t n = 0; n < stan::length(mu); ++n)

243  operands\_and\_partials.d\_x2[n] \*= P;

244  }

245  if (!is\_constant\_struct<T\_scale>::value) {

246  for(size\_t n = 0; n < stan::length(sigma); ++n)

247  operands\_and\_partials.d\_x3[n] \*= P;

248  }

249

250  return operands\_and\_partials.to\_var(P);

251  }

252

253  template <typename T\_y, typename T\_loc, typename T\_scale>

254  typename return\_type<T\_y,T\_loc,T\_scale>::type

255  cauchy\_cdf\_log(const T\_y& y, const T\_loc& mu, const T\_scale& sigma) {

256

257  // Size checks

258  if ( !( stan::length(y) && stan::length(mu)

259  && stan::length(sigma) ) )

260  return 0.0;

261

262  static const char\* function = "stan::prob::cauchy\_cdf(%1%)";

263

264  using stan::math::check\_positive\_finite;

265  using stan::math::check\_finite;

266  using stan::math::check\_not\_nan;

267  using stan::math::check\_consistent\_sizes;

268  using boost::math::tools::promote\_args;

269  using stan::math::value\_of;

270

271  double cdf\_log(0.0);

272

273  check\_not\_nan(function, y, "Random variable", &cdf\_log);

274  check\_finite(function, mu, "Location parameter", &cdf\_log);

275  check\_positive\_finite(function, sigma, "Scale parameter", &cdf\_log);

276  check\_consistent\_sizes(function, y, mu, sigma,

277  "Random variable", "Location parameter",

278  "Scale Parameter", &cdf\_log);

279

280  // Wrap arguments in vectors

281  VectorView<const T\_y> y\_vec(y);

282  VectorView<const T\_loc> mu\_vec(mu);

283  VectorView<const T\_scale> sigma\_vec(sigma);

284  size\_t N = max\_size(y, mu, sigma);

285

286  agrad::OperandsAndPartials<T\_y, T\_loc, T\_scale>

287  operands\_and\_partials(y, mu, sigma);

288

289  // Compute CDFLog and its gradients

290  using std::atan;

291  using stan::math::pi;

292

293  // Compute vectorized CDF and gradient

294  for (size\_t n = 0; n < N; n++) {

295

296  // Pull out values

297  const double y\_dbl = value\_of(y\_vec[n]);

298  const double mu\_dbl = value\_of(mu\_vec[n]);

299  const double sigma\_inv\_dbl = 1.0 / value\_of(sigma\_vec[n]);

300  const double sigma\_dbl = value\_of(sigma\_vec[n]);

301

302  const double z = (y\_dbl - mu\_dbl) \* sigma\_inv\_dbl;

303

304  // Compute

305  const double Pn = atan(z) / pi() + 0.5;

306  cdf\_log += log(Pn);

307

308  const double rep\_deriv = 1.0 / (pi() \* Pn

309  \* (z \* z \* sigma\_dbl + sigma\_dbl));

310  if (!is\_constant\_struct<T\_y>::value)

311  operands\_and\_partials.d\_x1[n] += rep\_deriv;

312  if (!is\_constant\_struct<T\_loc>::value)

313  operands\_and\_partials.d\_x2[n] -= rep\_deriv;

314  if (!is\_constant\_struct<T\_scale>::value)

315  operands\_and\_partials.d\_x3[n] -= rep\_deriv \* z;

316  }

317  return operands\_and\_partials.to\_var(cdf\_log);

318  }

319

320  template <typename T\_y, typename T\_loc, typename T\_scale>

321  typename return\_type<T\_y,T\_loc,T\_scale>::type

322  cauchy\_ccdf\_log(const T\_y& y, const T\_loc& mu, const T\_scale& sigma) {

323

324  // Size checks

325  if ( !( stan::length(y) && stan::length(mu)

326  && stan::length(sigma) ) )

327  return 0.0;

328

329  static const char\* function = "stan::prob::cauchy\_cdf(%1%)";

330

331  using stan::math::check\_positive\_finite;

332  using stan::math::check\_finite;

333  using stan::math::check\_not\_nan;

334  using stan::math::check\_consistent\_sizes;

335  using boost::math::tools::promote\_args;

336  using stan::math::value\_of;

337

338  double ccdf\_log(0.0);

339

340  check\_not\_nan(function, y, "Random variable", &ccdf\_log);

341  check\_finite(function, mu, "Location parameter", &ccdf\_log);

342  check\_positive\_finite(function, sigma, "Scale parameter", &ccdf\_log);

343  check\_consistent\_sizes(function, y, mu, sigma,

344  "Random variable", "Location parameter",

345  "Scale Parameter", &ccdf\_log);

346

347  // Wrap arguments in vectors

348  VectorView<const T\_y> y\_vec(y);

349  VectorView<const T\_loc> mu\_vec(mu);

350  VectorView<const T\_scale> sigma\_vec(sigma);

351  size\_t N = max\_size(y, mu, sigma);

352

353  agrad::OperandsAndPartials<T\_y, T\_loc, T\_scale>

354  operands\_and\_partials(y, mu, sigma);

355

356  // Compute CDFLog and its gradients

357  using std::atan;

358  using stan::math::pi;

359

360  // Compute vectorized CDF and gradient

361  for (size\_t n = 0; n < N; n++) {

362

363  // Pull out values

364  const double y\_dbl = value\_of(y\_vec[n]);

365  const double mu\_dbl = value\_of(mu\_vec[n]);

366  const double sigma\_inv\_dbl = 1.0 / value\_of(sigma\_vec[n]);

367  const double sigma\_dbl = value\_of(sigma\_vec[n]);

368  const double z = (y\_dbl - mu\_dbl) \* sigma\_inv\_dbl;

369

370  // Compute

371  const double Pn = 0.5 - atan(z) / pi();

372  ccdf\_log += log(Pn);

373

374  const double rep\_deriv = 1.0 / (Pn \* pi()

375  \* (z \* z \* sigma\_dbl + sigma\_dbl));

376  if (!is\_constant\_struct<T\_y>::value)

377  operands\_and\_partials.d\_x1[n] -= rep\_deriv;

378  if (!is\_constant\_struct<T\_loc>::value)

379  operands\_and\_partials.d\_x2[n] += rep\_deriv;

380  if (!is\_constant\_struct<T\_scale>::value)

381  operands\_and\_partials.d\_x3[n] += rep\_deriv \* z;

382  }

383  return operands\_and\_partials.to\_var(ccdf\_log);

384  }

385

386  template <class RNG>

387  inline double

388  cauchy\_rng(const double mu,

389  const double sigma,

390  RNG& rng) {

391  using boost::variate\_generator;

392  using boost::random::cauchy\_distribution;

393

394  static const char\* function = "stan::prob::cauchy\_rng(%1%)";

395

396  using stan::math::check\_positive\_finite;

397  using stan::math::check\_finite;

398

399  check\_finite(function, mu, "Location parameter", (double\*)0);

400  check\_positive\_finite(function, sigma, "Scale parameter", (double\*)0);

401

402  variate\_generator<RNG&, cauchy\_distribution<> >

403  cauchy\_rng(rng, cauchy\_distribution<>(mu, sigma));

404  return cauchy\_rng();

405  }

406  }

407 }

408 #endif

stan::math::square

T square(const T x)

Return the square of the specified argument.

**Definition:** square.hpp:22

stan::prob::cauchy\_rng

double cauchy\_rng(const double mu, const double sigma, RNG &rng)

**Definition:** cauchy.hpp:388

traits.hpp

stan::math::log1p

boost::math::tools::promote\_args< T >::type log1p(const T x)

Return the natural logarithm of one plus the specified value.

**Definition:** log1p.hpp:39

stan::agrad::OperandsAndPartials::to\_var

T\_return\_type to\_var(double logp)

**Definition:** partials\_vari.hpp:138

stan::math::check\_positive\_finite

bool check\_positive\_finite(const char \*function, const T\_y &y, const char \*name, T\_result \*result)

**Definition:** check\_positive\_finite.hpp:12

error\_handling.hpp

square.hpp

stan::length

size\_t length(const T &)

**Definition:** traits.hpp:159

stan::DoubleVectorView

DoubleVectorView allocates double values to be used as intermediate values.

**Definition:** traits.hpp:358

stan::math::check\_finite

bool check\_finite(const char \*function, const T\_y &y, const char \*name, T\_result \*result)

Checks if the variable y is finite.

**Definition:** check\_finite.hpp:52

stan::prob::cauchy\_cdf\_log

return\_type< T\_y, T\_loc, T\_scale >::type cauchy\_cdf\_log(const T\_y &y, const T\_loc &mu, const T\_scale &sigma)

**Definition:** cauchy.hpp:255

partials\_vari.hpp

stan::agrad::value\_of

T value\_of(const fvar< T > &v)

Return the value of the specified variable.

**Definition:** value\_of.hpp:16

constants.hpp

stan::is\_vector

**Definition:** traits.hpp:83

stan::agrad::OperandsAndPartials

A variable implementation that stores operands and derivatives with respect to the variable...

**Definition:** partials\_vari.hpp:76

stan::return\_type::type

boost::math::tools::promote\_args< typename scalar\_type< T1 >::type, typename scalar\_type< T2 >::type, typename scalar\_type< T3 >::type, typename scalar\_type< T4 >::type, typename scalar\_type< T5 >::type, typename scalar\_type< T6 >::type >::type type

**Definition:** traits.hpp:406

stan::agrad::atan

fvar< T > atan(const fvar< T > &x)

**Definition:** atan.hpp:16

stan::is\_constant\_struct

Metaprogram to determine if a type has a base scalar type that can be assigned to type double...

**Definition:** traits.hpp:57

stan::math::value\_of

double value\_of(const T x)

Return the value of the specified scalar argument converted to a double value.

**Definition:** value\_of.hpp:24

stan::prob::include\_summand

Template metaprogram to calculate whether a summand needs to be included in a proportional (log) prob...

**Definition:** traits.hpp:35

stan::agrad::OperandsAndPartials::d\_x2

VectorView< double \*, is\_vector< T2 >::value, is\_constant\_struct< T2 >::value > d\_x2

**Definition:** partials\_vari.hpp:83

stan::prob::cauchy\_ccdf\_log

return\_type< T\_y, T\_loc, T\_scale >::type cauchy\_ccdf\_log(const T\_y &y, const T\_loc &mu, const T\_scale &sigma)

**Definition:** cauchy.hpp:322

stan::math::check\_consistent\_sizes

bool check\_consistent\_sizes(const char \*function, const T1 &x1, const T2 &x2, const char \*name1, const char \*name2, T\_result \*result)

**Definition:** check\_consistent\_sizes.hpp:13

stan::max\_size

size\_t max\_size(const T1 &x1, const T2 &x2)

**Definition:** traits.hpp:191

stan::prob::cauchy\_log

return\_type< T\_y, T\_loc, T\_scale >::type cauchy\_log(const T\_y &y, const T\_loc &mu, const T\_scale &sigma)

The log of the Cauchy density for the specified scalar(s) given the specified location parameter(s) a...

**Definition:** cauchy.hpp:42

log1p.hpp

stan::math::check\_not\_nan

bool check\_not\_nan(const char \*function, const T\_y &y, const char \*name, T\_result \*result)

Checks if the variable y is nan.

**Definition:** check\_not\_nan.hpp:56

value\_of.hpp

stan::agrad::OperandsAndPartials::d\_x1

VectorView< double \*, is\_vector< T1 >::value, is\_constant\_struct< T1 >::value > d\_x1

**Definition:** partials\_vari.hpp:82

stan::agrad::OperandsAndPartials::d\_x3

VectorView< double \*, is\_vector< T3 >::value, is\_constant\_struct< T3 >::value > d\_x3

**Definition:** partials\_vari.hpp:84

stan::math::pi

double pi()

Return the value of pi.

**Definition:** constants.hpp:77

constants.hpp

stan::agrad::log

fvar< T > log(const fvar< T > &x)

**Definition:** log.hpp:15

stan::VectorView

VectorView is a template metaprogram that takes its argument and allows it to be used like a vector...

**Definition:** traits.hpp:275

stan::agrad::log1p

fvar< T > log1p(const fvar< T > &x)

**Definition:** log1p.hpp:16

stan::prob::cauchy\_cdf

return\_type< T\_y, T\_loc, T\_scale >::type cauchy\_cdf(const T\_y &y, const T\_loc &mu, const T\_scale &sigma)

Calculates the cauchy cumulative distribution function for the given variate, location, and scale.

**Definition:** cauchy.hpp:158


---

[ Stan Home Page ]

*© 2011–2014,
Stan Development Team.*
